# The British Journals

**Published:** 1840-07

**Authors:** 


### III. THE BRITISH JOURNALS.

(FOR THE QUARTER ENDING MAY 31, 1840.)

[Owing to the extent of our other materials this quarter, we are forced to content ourselves with little more than the titles of some of the more valuable papers.—Ed.]

### Edinburgh Medical and Surgical Journal. April, 1840. No. 143.

*Historical Notices, designed to illustrate the question whether Typhus ought to be classed among the Exanthematous Fevers.* By Charles West, m.d., Graduate of the University of Berlin.

This is a sensible and well-written paper, "the aim of which is to disprove the claim of typhus fever to a place among the true eruptive fevers." The conclusions to which the author has arrived are, perhaps, the more worthy of attention that he began his investigations with a strong prepossession in favour of the opposite view. The following are the chief facts on which he grounds his opinions: 1st. The disease often occurs more than once during the lifetime of an individual. 2d. The eruption is not invariably present. 3d. The eruption does not always present the same character, nor does it always run a regular course, observing definite periods of increase, acme, and decline. 4th. The type of the fever itself varies, being sometimes intermittent, sometimes continued, changing from the one to the other form, and being occasionally converted into other diseases.

---

*Practical Observations on the Diseases of Peru, described as they occur on the Coast and in the Sierra.* By Archibald Smith, m.d.

A valuable contribution to topographical medicine.

---

*Remarks on Collapse occurring during the treatment of Acute Pneumonic Diseases.* By William Kerr, Surgeon, Paisley.

The perusal of these cases will benefit inexperienced practitioners. They are candidly related.

---

*An Account of the Rötheln of German Authors, together with a few Observations on the Disease as it has been seen to prevail in Leith and its neighbourhood.* By Robert Paterson, m.d. &c., Physician to the Leith Dispensary.

This affection has generally been confounded with measles, sometimes with scarlatina. Dr. Paterson considers it intermediate between the two. This disease is characterized by an eruption of crimson stigmata, rapidly running together into patches of an irregular shape, with obtuse angles, varying much in size (from that of a pea to a crown-piece) according to the severity of the case, being smaller in mild and larger in severer cases; continuing for six or ten days, and terminating in furfuraceous scales; preceded by and accompanied with fever; watery discharges from eyes and nose; sneezing, and sore throat.

---

### Dublin Journal. March—May, 1840. Nos. 49, 50.

*A further Reply to Mr. H. Carmichael's Views on the Position of the Placenta, &c., in which is given a Statistical Table of its Situation in 100 Cases, as determined by the Stethoscope and the Measurements of the Membranes.* By Richard Doherty, m.d.

We much regret that we cannot transfer to our pages the important table given in this valuable paper, which besides exhibiting on a large scale the actual position of the placenta, places in a striking light the value of obstetric auscultation. The table proves that out of these 100 cases, in twenty-five the placenta was attached to the anterior wall; in eight to the right side, below the Fallopian tubes; in ten to the left side, below the tubes; in three to the fundus; in fifty-four to the posterior wall; and of these only twenty-seven came "within two inches of the lowest part of the cyst." It proves also what has been doubted by some writers, that the stethoscope is capable of pointing out the exact situation of the placenta, a point which may be of service, if we afterwards are obliged artificially to remove it. A knowledge of its position will direct us which hand to employ, and enable us to pass it without hesitation in the proper direction, separating the membranes from the uterus as we proceed, and thus obviating the embarrassment they often afford while detaching a morbidly adherent placenta. No. 49. *March.*

---

*Practical Observations on Peculiar Affections of the Throat, arising from Abscess between the Pharynx and Spine, and occurring in Children and Adults, exemplified by Cases.* By CHRISTOPHER FLEMING, M.D., Member of the Royal College of Surgeons, Ireland.

THIS is a valuable communication, and well merits the attention of physicians as well as surgeons, as the affection therein noticed may be very readily overlooked, and probably often is so to the loss of life. *Ibid.*

---

*On Retroversion of the Uterus, and a new Method of treating that Affection.* By CHARLES HALPIN, Esq. of Cavan, L.R.C.S. in Ireland.

THIS paper is worth consulting. The "new method" consists in the introduction of an empty bladder, and then inflating it, so as to produce thereby "a steady, equal, well-directed pressure" on the retroverted uterus, and thereby restoring it to its proper position. *Ibid.*

---

*Appendix to Dr. Graves's Report on the Progress of Cholera, including the Tables from the Official Report of the Commissioners appointed to report the Progress of Cholera in Great Britain.*

THESE tables are taken from the official report presented by the Commissioners to William IV., of which report but one copy existed; that copy was mislaid, and would have been lost to the world, had not Sir James Clark exerted himself to search it out, and by the aid of the Royal Librarian it was at last found buried amongst an heterogeneous mass of papers in a drawer.

These tables are valuable in the highest degree, and can never be overlooked in any future history of cholera. The commissioners performed their duty with a diligence and accuracy which stand unrivalled, and their labours have succeeded in furnishing a more valuable and accurate history of the march and effects of cholera than any other country can boast. By the aid of these tables, any person can construct a map of the progress of cholera in Great Britain, which will at once exhibit its route and intensity. *Ibid.*

---

*Observations on Plastic Bronchitis, or Bronchial Polypi.* By ROBERT CANE, M.D. M.R.C.S.L., Kilkenny.

Two well-marked cases of this affection, and both terminating favorably under the bold use of mercury. The treatment deserves attention. *Ibid.*

---

*Disease of the Brain dependent on Disease of the Heart.* By ROBERT LAW, M.D., Physician in Ordinary to Sir Patrick Dun's Hospital, &c. &c.

THIS paper deserves notice. The author justly considers that to limit the pathological relation existing between these two important organs to apoplexy, the result of hypertrophy of the left ventricle of the heart, is to narrow it much within its true limits. He takes the same view as Dr. Carswell of softening of the brain, viz. that it is a species of gangrene consequent upon a diminution of a due supply of blood; but he considers that this diminution may depend on disease of the heart as well as of the cerebral vessels, as, for instance, on disease of the aortic or mitral valve. No. 50. *May.*

---

*Observations on Empyema.* By GEORGE GREENE, M.D., Lecturer on the Practice of Medicine in the Richmond Hospital School, Dublin.

THESE observations are deserving attention. They contain some new facts respecting the expectoration in empyema, which have an important bearing on the diagnosis both of empyema and phthisis, and on the propriety of the surgical operation in the former. *Ibid.*

---

*Smallpox Pustule in the Bladder.*

DR. GREENE presented a remarkable specimen of smallpox on the mucous membrane of the bladder. The patient, a young man, died of smallpox. While convalescent from fever, he caught infection from another person in the same room, and had the disease in the confluent form. He died on the fifth day after the appearance of the eruption, and shortly before his death was attacked with severe diarrhœa. There were no pustules in the respiratory passages or intestinal tube. *Ibid.*

---

### LANCET. MARCH—MAY, 1840.

*Treatment of Disease of the Hip-joint by Salivation with Mercury.*
By JAMES O'BEIRNE, M.D. Surgeon Extraordinary to the Queen.

DR. O'BEIRNE has been in the habit for some years of treating morbus coxæ most successfully by rapidly mercurializing the system by means of calomel and opium. In addition to this means he confines the patient to the horizontal position and gives sarsaparilla. He avoids purgatives and leeches, and all kinds of counter-irritation until ptyalism is established. This practice is a great improvement in our therapeutics. *March* 7.

---

*On the Hydrated Sesquioxide of Iron as an Antidote to Arsenic.*
By DONALD MACKENZIE, M.D.

FROM seven experiments on dogs here related, Dr. Mackenzie concludes that the hydrated sesquioxide of iron should be resorted to in all cases where arsenic has been swallowed, as it is a chemical antidote of great avail in poisoning by that substance. *April* 4.

---

*On a New Mode of Treating Incontinence of Urine during Sleep.*
By E. W. DUFFIN, Esq., Surgeon, London.

THIS consists in the application of lunar caustic to the orifice of the urethra, so as to excite acute inflammation of the part; a case is given of its utility. "When the urine passed over the irritated surface the pain it produced was sufficient to awaken the patient, and arouse the sphincter vesicæ to the performance of its office." The caustic was repeated and a cure shortly effected. *April* 11.

---

*Cure for Corns.* By JAMES HENDERSON, Surgeon.

Tincture of Iodine, ℥iv.;
Ioduret of Iron, xij. gr.;
Chloride of Antimony, ℥iv.;

To be applied by means of a camel hair brush, after the corn has been well pared. Three applications have generally been found sufficient. *April* 8.

*Cases illustrating the efficacy of Dividing the Internal Rectus Muscle for the Cure of Squinting.* By P. Bennett Lucas, Esq.

Mr. Lucas has performed this operation in five cases with perfect success, and, we believe, he was the first to perform it in England. Mr. Lucas has somewhat simplified Dieffenbach's method, employing only one hook in place of four. *May* 2.

---

*Cure of Squinting.* By Mr. Liston.

Mr. Liston has also performed this operation several times and with the usual success; his method is the following:

"The lower eyelid is everted, and with a pair of spring artery forceps a small portion of the conjunctiva is seized; the forceps are then allowed to hang down, and the eyelid is thus held completely open; the upper eyelid is held up with the common speculum; a small double hook is placed in the conjunctiva, internally to the cornea, and the eye is pulled outwards; the conjunctiva being snipped across with scissors, and the sclerotic exposed, another hook is placed in this membrane, and the eye more forcibly everted. With a little dissection the muscle is seen just as it ends in its tendon, and with a pair of scissors it is cut across close to its insertion into the sclerotic; the operation does not occupy half a minute when the patient is quiet, and one assistant only is required." *May* 23.

---

### London Medical Gazette. March—May, 1840.

*Observations on the Employment of the Active Principle of Elaterium in Medicine.* By Golding Bird, m.d. f.l.s., &c.

This is a valuable paper, showing that *elaterine* ought always to be preferred to the crude drug. "I have had," says Dr. Bird, "a sufficient amount of experience to justify my recommending this drug to the notice of my professional brethren as a valuable addition to our materia medica, not as a new or specific remedy, but simply as an hydragogue cathartic, producing little or no irritation; if properly administered, not liable to the annoying inconstancy of action which characterizes the crude elaterium, and not producing vomiting or griping, unless considerable functional gastric or hepatic derangement is present." *March* 13.

---

*On the Frequency of the Pulse at Different Ages in Males and Females.* By W. Aug. Guy, m.b., Cantab, Prof. of Forensic Medicine, King's Col., London.

A continuation of the author's valuable researches on this important subject.

---

*Acetate of Lead in Bronchitis.* By W. Henderson, m.d.

After a careful investigation of the powers of this remedy, continued for several years, Dr. Henderson feels warranted in stating his conviction that the acetate of lead is a remedy by far the most worthy of reliance in bronchitis attended with profuse secretion. Its administration has been limited to that period of the bronchitis in which the evidences of abundant secretion were apparent; and those evidences have formed the only guides which he has found it requisite to follow in the first exhibition of the remedy, and in regulating the bulk and frequency of the doses. The dose has been, for children, gr. ¼, gr. ss, gr. j, eight or ten times a day, and from one to three grains for an adult, not exceeding in all twelve grains a day. *May* 8.

---

*Account of Laura Bridgman, an American Girl, with only one Sense.* By Dr. Julius, of Hamburg.

This is a very interesting narrative. The subject of it is a young girl, nine years of age. In addition to her deafness, dumbness, and blindness, her exclusion from the external world is so entire, that she is almost destitute of smell, and can only smell the most pungent things. And yet she is intelligent, and has made considerable progress in gaining information. *May* 15.

